# Serum metabolomics profiles in response to n-3 fatty acids in Chinese patients with type 2 diabetes: a double-blind randomised controlled trial

**DOI:** 10.1038/srep29522

**Published:** 2016-07-12

**Authors:** Ju-Sheng Zheng, Mei Lin, Fumiaki Imamura, Wenwen Cai, Ling Wang, Jue-Ping Feng, Yue Ruan, Jun Tang, Fenglei Wang, Hong Yang, Duo Li

**Affiliations:** 1 Department of Food Science and Nutrition, Zhejiang University, Hangzhou 310058, China; 2 Medical Research Council Epidemiology Unit, University of Cambridge School of Clinical Medicine, Cambridge, Institute of Metabolic Science, Cambridge Biomedical Campus, Cambridge CB2 0QQ, UK; 3 Department of Endocrinology, Wuhan Puai Hospital of Tongji Medical College, Huazhong University of Science and Technology, Wuhan, 430034, China; 4 College of Food Science and Technology, Huazhong Agricultural University, Wuhan 430070, China; 5 Wellcome Trust-MRC Institute of Metabolic Science, University of Cambridge, Cambridge, CB2 0QQ, UK; 6 Department of Paediatrics, University of Cambridge, Cambridge, CB2 0QQ, UK

## Abstract

We aimed to investigate the change of serum metabolomics in response to n-3 fatty acid supplements in Chinese patients with type 2 diabetes (T2D). In a double-blind parallel randomised controlled trial, 59 Chinese T2D patients were randomised to receive either fish oil (FO), flaxseed oil (FSO) or corn oil capsules (CO, served as a control group) and followed up for 180 days. An additional 17 healthy non-T2D participants were recruited at baseline for cross-sectional comparison between cases and non-cases. A total of 296 serum metabolites were measured among healthy controls and T2D patients before and after the intervention. Serum 3-carboxy-4-methyl-5-propyl-2-furanpropanoate (CMPF) (*P*-interaction = 1.8 × 10^−7^) was the most significant metabolite identified by repeated-measures ANOVA, followed by eicosapentaenoate (*P*-interaction = 4.6 × 10^−6^), 1-eicosapentaenoylglycerophosphocholine (*P*-interaction = 3.4 × 10^−4^), docosahexaenoate (*P*-interaction = 0.001), linolenate (n-3 or n-6, *P*-interaction = 0.005) and docosapentaenoate (n-3, *P*-interaction = 0.021). CMPF level was lower in T2D patients than in the healthy controls (*P* = 0.014) and it was significantly increased in the FO compared with CO group (*P* = 1.17 × 10^−7^). Furthermore, change of CMPF during the intervention was negatively correlated with change of serum triglycerides (*P* = 0.016). In conclusion, furan fatty acid metabolite CMPF was the strongest biomarker of fish oil intake. The association of CMPF with metabolic markers warrants further investigation.

Type 2 diabetes (T2D) is characterized by insulin resistance and impaired β-cell function. These manifestations involve complex biological pathways, partly evidenced by metabolomics studies^1^,^2^,^3^. Amino acids, sugar metabolites and choline-containing phospholipids have been identified to be of potential importance in the pathogenesis of T2D^4^,^5^,^6^,^7^, in addition to traditional biomarkers, such as fasting glucose and adiponectin.

The human metabolome contains a huge number of molecules derived from diet. Thus, metabolomics is a promising approach to explain pathophysiological roles of dietary components. Long-chain n-3 polyunsaturated fatty acids (PUFA) are of interest topic, because of inconsistent findings in literature. Animal studies have provided convincing evidence that n-3 PUFA improve glucose metabolism^8^,^9^, with a variety of potential mechanisms including improved membrane fluidity^8^, anti-inflammatory effect^10^, and genetic regulation^11^. Despite the biological plausibility, studies in humans have yielded conflicting results. Meta-analyses based on observational studies show that dietary intakes of long-chain n-3 PUFA are inversely associated with T2D risk in Asian populations, but positively associated with the risk in Western populations^12^,^13^. Randomised controlled trials indicate no significant effect of long-chain n-3 PUFA on glucose-insulin homeostasis^14^,^15^, although n-3 PUFA substantially reduce blood concentrations of triglycerides (TG), one of risk factors for T2D and cardiovascular diseases^16^,^17^. To explain the conflict between mechanistic studies and human studies and to better understand how n-3 PUFA might influence the pathogenesis of T2D, metabolomics profiles in response to n-3 PUFA intervention in T2D patients are warranted.

We conducted a multi-centre double-blind parallel randomised controlled trial (n = 185) to investigated the effects of n-3 PUFA supplements (both marine [fish oil] and plant source [flaxseed oil]) on glycaemic traits in Chinese patients with T2D. This trial found that fish oil supplement, compared with corn oil control group, significantly decreased glycated hemoglobin (HbA1c) and TG^18^. In the present study, using serum metabolomics data (n = 53) from a single centre of the aforementioned multi-centre trial (Fig. 1), our primary aim was to examine change of serum metabolomics in response to n-3 fatty acid supplements in Chinese T2D patients; and the secondary aims were to conduct a case-control comparison for serum metabolomics between T2D cases and non-cases, and to examine the correlation of the serum metabolites with metabolic markers among T2D cases and non-cases.

## Results

### Characteristics of the study population and compliance of the participants

Among the three intervention groups, no significant difference in anthropometric or biochemical parameters was observed at baseline (Table 1). However, FO group showed fewer women participants, lower BMI, TG and HDL-C than CO group. The healthy controls, compared with T2D patients in the trial, had significantly lower BMI, systolic and diastolic blood pressure, LDL-C and fasting glucose, and higher HDL-C concentration (*P* < 0.05) (Table 1). Changes of blood metabolic markers were presented in the Supplemental file (Supplemental file 1: Supplemental Table 1).

As a test of compliance to the intervention, erythrocyte eicosapentaenoic acid (EPA, C20:5n3) and docosahexaenoic acid (DHA, C22:6n3) significantly increased from 0.59% (standard error [SE] = 0.05) and 4.86% (SE = 0.26) to 1.65% (SE = 0.32) and 6.55% (SE = 0.34) respectively in the fish oil (FO) arm, compared with the change from 0.44 (SE = 0.04) to 0.41 (SE = 0.03) in the corn oil (CO) arm (*P* = 0.035 for EPA, *P* = 0.001 for DHA). Erythrocyte alpha-linolenate (ALA, C18:3n3) levels were increased in the FSO arm, but not significantly different from change in the CO arm (*P* = 0.13).

### Change of serum metabolites in response to n-3 PUFA intervention

#### Results of pattern recognition analyses: principal component analysis (PCA) and partial least square-discriminant analysis (PLS-DA)

For PCA, five principal components were derived, as linear combinations of changes in metabolites: the number of components was based on scree plot and the five components explained 35% of total variability of metabolites’ changes. The first principal component was significantly different between the FSO arm and the CO arm (*P* = 0.006). No significant difference between different groups was observed for the other components. Of 296 metabolites, 30 metabolites, the most contributing to the first principal component, were selected for visualization (Supplemental file 1: Supplemental Figure 1), including fatty acid species and derivatives with various chain lengths such as palmitate, linoleate, acyl-carnitines, and hydroxyl fatty acids. For PLS-DA, we found that all of the top three components could significantly (*P* < 0.05) separate FO and FSO group from CO group (Supplemental file 1: Supplemental Table 2). With ranking by the variable importance in projection (VIP) score, 3-carboxy-4-methyl-5-propyl-2-furanpropanoate (CMPF) was the top metabolite identified for the component distinguishing the FO and CO groups, followed by PC-EPA (1-eicosapentaenoylglycerophosphocholine) and EPA (Supplemental file 1: Supplemental Figure 2A). For the distinction between FSO and CO groups, indolepropinate, erucate and mannitol were the top three metabolites identified by the ranking (Supplemental file 1: Supplemental Figure 2B).

#### Results from repeated-measures ANOVA.

Changes of six metabolites were significantly different between trial arms (Table 2). CMPF showed the lowest *P*-value of 1.8 × 10^−7^ for interaction of time and groups, followed by serum EPA (*P* = 4.6 × 10^−6^), PC-EPA (*P* = 3.4 × 10^−4^), DHA (*P* = 0.001), linolenate (C18:3n-3 or n-6, *P* = 0.005) and n-3 DPA (docosapentaenoate, C22:5n-3) (*P* = 0.021). In post hoc analysis comparing between FO and CO groups, FO group showed significant increases in concentrations of CMPF (*P* = 1.17 × 10^−7^ for time × group interaction), EPA (*P* = 5.81 × 10^−6^), PC-EPA (*P* = 8.21 × 10^−5^), DHA (*P* = 1.79 × 10^−4^) and DPA (*P* = 8.27 × 10^−4^), while the CO group showed trends toward declines of these PUFA’ concentrations. For the comparison between FSO and CO groups, linolenate (*P* = 7.75 × 10^−6^), n-3 DPA (*P* = 3.57 × 10^−4^), EPA (*P* = 4.68 × 10^−4^) and PC-EPA (*P* = 0.008) showed significant time × group interaction.

### Metabolomics profiles of T2D patients and healthy controls

Of the 296 metabolites, 151 were significantly different between T2D patients and healthy controls at baseline after adjustment for age, sex and BMI and correction for multiple testing, among which 99 were lower in T2D patients than in healthy controls and 52 higher (Supplemental file 1: Supplemental Table 3, Supplemental Figure 3). We found that CMPF (*P* = 0.014), PC-EPA (*P* = 5.49 × 10^−6^), EPA (*P* = 2.45 × 10^−7^) and DHA (*P* = 4.22 × 10^−7^) in T2D patients at baseline was significantly lower than in healthy controls (Supplemental file 1: Supplemental Figure 4).

### Correlation of furan fatty acid metabolite CMPF with serum n-3 fatty acids and metabolic traits

In the analysis of correlations between metabolites in those with or without diabetes together at baseline, CMPF showed positive association with serum EPA (*r* = 0.27, *P* = 0.031, adjusted for age, sex, BMI, and diabetes status) and DHA (*r* = 0.31, *P* = 0.013), but not with other metabolic markers. In analysis among T2D patients in the trial, the change of CMPF was correlated with those of serum EPA (*r* = 0.62, *P* < 0.001) and DHA (*r* = 0.57, *P* < 0.001) (Fig. 2); and not significantly correlated with any other examined metabolic risk factor, except for TG (*r* = −0.35, *P* = 0.016) (Fig. 2). This inverse correlation between changes of CMPF and TG remained significant with further adjustment for the change of serum EPA and DHA (*r* = −0.31, *P* = 0.039).

## Discussion

To the best of our knowledge, this was the first study to investigate the metabolomics change in response to randomised controlled interventions of plant-based and marine-based n-3 PUFA in Chinese T2D patients. Pattern-recognition approach showed that both the plant-based and marine-based PUFA interventions led to significant changes in metabolite patterns related to medium-chain and long-chain fatty acids. We found that the concentration of CMPF the most significantly increased in response to the intervention of marine-based PUFA. In the case-control comparison at baseline, CMPF was substantially higher in the healthy controls than in the T2D patients. Furthermore, change of CMPF during the intervention was negatively correlated with change of serum TG, which appeared to be independent of change of serum n-3 PUFA.

CMPF has been studied for its potential adverse effect on glucose metabolism. CMPF is a major endogenous metabolite of furan fatty acids in human, and it mainly derived from fish consumption or potentially from oxidation of unsaturated fatty acids^19^,^20^,^21^. In a recent study, CMPF was found to be elevated in the plasma of patients with gestational diabetes, impaired glucose-tolerance or T2D^22^. In addition, a rodent experiment demonstrated that CMPF could directly affect β-cell function, impair mitochondrial function, decrease glucose-induced ATP accumulation and induce oxidative stress^22^. In addition, plasma CMPF concentration in 106 non-diabetic Finnish participants of a recent randomised trial was much lower than diabetic patients^23^. Because of the potential adverse effect of CMPF on diabetes outcomes, higher levels of CMPF in T2D patients than in healthy controls would be expected. However, opposite result was observed in the present study in China.

Given that circulating CMPF was a potential biomarker of fish oil supplements and that circulating marine n-3 PUFA and CMPF were high correlated, it was reasonable to postulate that the association of CMPF with metabolic markers may be similar as to that of marine n-3 PUFA. In line with this postulation, available evidence suggested that the inconsistent findings for the associations of CMPF with glycaemic traits and T2D status were similar to prior findings for marine n-3 PUFA. For example, Prentice *et al*.^2^ reported that plasma marine n-3 PUFA concentration was higher in diabetic patients compared with the controls. In contrast, our previous work in China showed that marine n-3 PUFA was lower in T2D patients compared with controls^24^. Inconsistency related to n-3 PUFA between Western and Asian populations was observed in prospective cohort studies: consumption of marine n-3 PUFA was inversely associated with T2D risk in Asian populations, but not in the Western populations^13^,^25^. Because sample sizes in the present study and the previous investigations on CMPF were small^22^,^23^, a study with a larger sample size both in Asian and Western populations is needed to further explore the role of CMPF in the etiology of T2D. In addition, detailed relationship of CMPF with n-3 PUFA metabolism is not clear at this stage and warrant further investigation.

Circulating CMPF was elevated after fish or fish oil consumption, as shown in the present study and previous trials^20^,^23^,^26^, possibly reflecting that EPA and furan fatty acid, precursors of CMPF^20^, were present in fish or fish oil. One interesting finding in this study was that CMPF was identified as the strongest correlate with fish oil intervention. Moreover, we found that increased serum CMPF was significantly associated with decreased serum TG, an important risk factor for diabetes and cardiovascular diseases. In contrast, increased serum concentration of CMPF was not significantly correlated with any glycaemic parameters in the present study, which was consistent with trials of fish oil supplements^14^,^27^. Therefore, the overall association of CMPF with glycaemic traits was not clear, and study with larger sample size is needed for replication. Another point of concern is that CMPF is considered as a protein-bound uremic toxin, which is elevated in patients with chronic kidney disease and demonstrated to be detrimental for renal function^28^,^29^. Therefore, at least with foci on cardiovascular health, diabetes, and kidney function, future research on CMPF is necessary. Future research on CMPF should evaluate its effects on different tissues and physiological functions.

We did not observe significant difference in change for the erythrocyte ALA composition between FSO and CO groups; however, we found that the metabolites’ pattern characterized by PCA distinguished FSO and CO group as visualized partly with a cluster tree of 30 metabolites. Most of these 30 metabolites were fatty acids and derivatives, which indicated that an FSO supplement may remarkably change multiple fatty acid metabolites. The possible mechanism is that various PUFA in FSO influence fatty acid metabolism in the liver rather than individual fatty acids, for example suppressing fatty acid synthesis and further triglycerides synthesis^30^. This observation highlights needs for further investigation.

Major limitations of the present study are the small sample size, the number of statistical tests that might include false-positive findings and that more than half of the patients were taking statin, which may influence lipid metabolism. Apparently, replication in independent populations is needed. Yet it is the first study to investigate the metabolomics response to plant-based and marine-based n-3 PUFA in T2D patients with the randomised controlled design and to provide novel information on CMPF. Another limitation is that the available metabolites examined in the study are still insufficient to fully understand the effects of n-3 PUFA supplements on lipid and glucose metabolism. Furthermore, the compliance to the intervention and to fasting hours may vary among different groups, although erythrocyte fatty acid composition measurement before and after the trial has some suggestions for the compliance. Last, the baseline characteristics (such as sex, BMI, TG, HDL-C) among different trial arms may different from each other, although no significant difference was detected (due to small sample size). The potential unbalanced baseline features was consistent with that of serum metabolite linolenate among the three trial arms, which was much lower in FSO group compared with the other groups. These unbalanced baseline characteristics may potentially bias the results of the present study.

In conclusion, the present study suggested that serum furan fatty acid metabolite CMPF was the strongest biomarker of fish oil intake, and change of serum CMPF was negatively correlated with change of serum TG in Chinese T2D patients. These finding could potentially spark interest among scientists to further investigate the role of CMPF in the n-3 PUFA-T2D association, as well as its role in lipid metabolism and non-communicable diseases.

## Methods

### Participants and study design

The current study evaluated metabolomics data from 53 adults with diabetes who participated in Wuhan, China, one of sites of a multi-centre trial in China; and 17 controls without diabetes who underwent health examination. The trial protocol was registered and available at ClinicalTrials.gov (No. NCT01857167) on May 10, 2013. The study was approved by the Ethics Committee of College of Biosystem Engineering and Food Science at Zhejiang University (No. 2013011). All the participants gave written informed consent. The study methods and reporting were carried out in accordance with the CONSORT 2010 guidelines.

The primary aim of the multi-centre randomised controlled trial was to investigate the effect of n-3 PUFA suppelments on glycaemic traits in Chinese T2D patients. The trial screened 252 potentially-eligible adults with known T2D status in three study centres from June 2013 to June 2014 and recruited 185 T2D cases: Wuhan (Central China) (n = 59, assessed for metabolomics), Changshan (Southeast China) (n = 47) and Lanzhou (Western China) (n = 79). The total sample size was calculated based on 80% power (α_two-tailed_ = 0.05) to detect difference in homeostasis model assessment for insulin resistance (HOMA-IR) by 20% or 0.63 (SD = 1.1) between groups, with consideration of 20% drop-out rate (n = 187, 62/group), based on our previous work^31^. The inclusion criteria were (1) fasting blood glucose >7.0 mmol/L or on use of diabetic medications; (2) between 35 and 80 years old for men and between post-menopausal and 80 years old for women. The exclusion criteria were (1) having familial hypertriglyceridemia or with blood TG concentrations >4.56 mmol/L; (2) a history of hepatic or kidney disease, or any type of cancer; (3) participation in another clinical trial within 30 days prior to screening.

After screening, participants were randomly assigned to one of the three groups by computer-generated random numbers with a block size of six: fish oil (FO, 4 capsules/day, 50% of EPA and DHA), flaxseed oil (FSO, 4 capsules/day, 63% of ALA) and corn oil (CO, 4 capsules/day, served as a control within this trial). Allocation sequence was generated by Ju-Sheng Zheng, and doctors/nurses at each study center enrolled and assigned participants to the intervention groups. The dosage levels of n-3 PUFA were consistent with those used in previous trials^32^. The duration of intervention was 180 days. All the participants were given four bottles of capsules (90 capsules/bottle) for 90-day intervention at baseline and the mid-point of the intervention (90 days). Types of capsules were concealed. At visit of 90 days and the end-point of intervention, participants underwent on-site examination.

**Figure 1:**
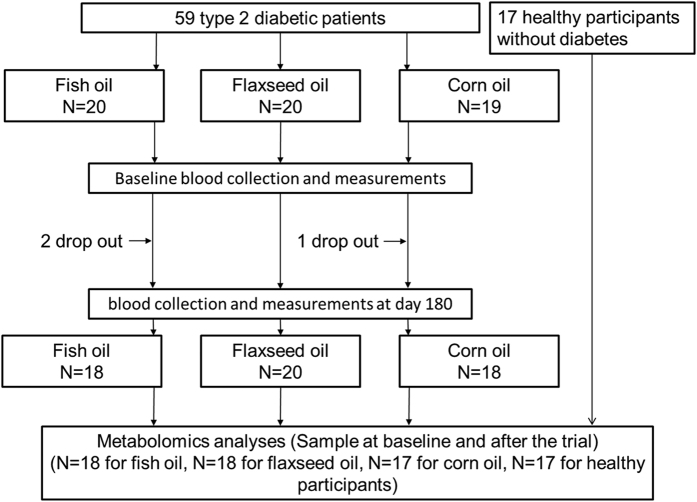
Design of the present study.

**Figure 2:**
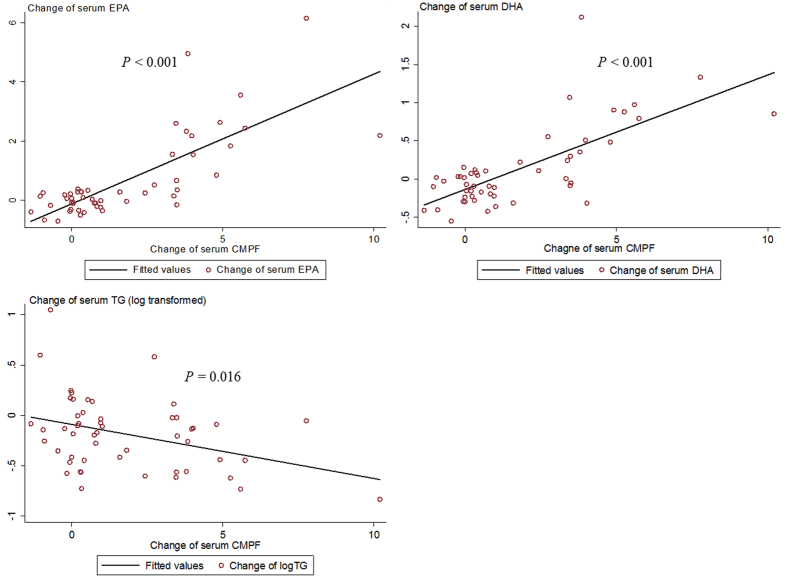
Correlation between changes of CMPF with changes of serum EPA, DHA and TG during the intervention. *P*-values were adjusted for age, sex, BMI and trial arms. The inverse correlation between changes of CMPF and TG remained significant with further adjustment for the change of serum EPA and DHA (*r* = −0.31, *P* = 0.039). EPA, eicosapentaenoate; DHA, docosahexaenoate. TG, triglycerides; CMPF, 3-carboxy-4-methyl-5-propyl-2-furanpropanoate.

In the present study based on a single centre in Wuhan, China (n = 59), participants were randomly allocated to FO (n = 20), FSO (n = 20) and CO group (n = 19). Three participants (two in FO group and one in CO group) dropped out of the trial. Among the remaining 56 participants, serum samples of 53 participants were assayed for metabolomics profiling (Fig. 1). Compliance to the intervention was assessed by repeated measurements of erythrocyte membrane phospholipid n-3 fatty acid compositions. Among the 53 participants, 29 of them were taking statins.

In the single centre, a case-control study was simultaneously conducted to compare the metabolomics profiles between T2D cases and non-cases. Seventeen healthy controls were recruited through a health-check program undertaken in the same period as the trial ran. They were not invited to the trial and were informed of the aim of the observational study. The inclusion and exclusion criteria were the same as the criteria applied to the above trial, except for the T2D case status.

### Experiment oil capsule preparation

We standardized each of fish oil (FO), flaxseed oil (FSO) or corn oil (CO) capsules to one gram with identical appearance. Each FO capsule provided 500mg of EPA + DHA (EPA: DHA = 3:2), and other major fatty acids in each FO capsule were C16:0 (71.4 mg), C18:1n-9 (58.4 mg), C16:1 (56 mg), C20:0 (39.4 mg) and C14:0 (34.6 mg). Each FSO capsule contain 630 mg of ALA, 155 mg of C18:2n-6 and 137 mg of C18:1n-9. Major fatty acids in each CO capsule were C18:2n-6 (534 mg), C18:1n-9 (299 mg) and C16:0 (121 mg). All the capsules were made in the Neptunus Bioengineering Co., Ltd (Hangzhou, China). All the capsules were kept in white bottles (90 capsules/bottle), which were labeled as Oil A, Oil B and Oil C for the three types of capsules. None of the participants or the nurses/physicians in the study centers knew the oil types during the intervention.

### Serum metabolomics measurement

Venous blood samples were collected from participants in this trial and case-control study (n = 76 in total) after an overnight fasting (>8 h): for trial participant, follow-up samples were also collected. Sera were shipped on dry ice to Zhejiang University, Hangzhou, China, for storage at −80 °C. Then, 500 μl of sera were shipped to Metabolon’s Shanghai Jiao Tong University Laboratory (Shanghai, China) for metabolomics assays with an non-targeted gas-chromatography- and liquid chromatography-mass-spectrometry^33^,^34^. This method identified 407 signals confirmed with molecular mass detected with either positive or negative ion mode, including CMPF, phospholipid species, and fatty acid acyl chains of DHA, EPA, and other fatty acids. Single molecules or fragments were evaluated as individual metabolites, expressed as relative strengths of signals across study participants (trial participants and controls). The details of the assays and quality-control analyses, conducted by Metabolon, Inc. (Durham, North Carolina), are presented in the Supplemental file 2.

### Measurement of anthropometric and blood biochemical parameters

Heart rate, blood pressure and anthropometric parameters, including weight, height, waist and hip circumference, were measured by trained staff at baseline, 90 days and 180 days of the intervention. Serum biochemical parameters were assessed at the baseline and endpoint of the trial with samples randomly distributed over assays, where assessors were blinded for types of oils. High-density lipoprotein cholesterol (HDL-C), low-density lipoprotein cholesterol (LDL-C), total cholesterol, TG and glucose levels were measured by commercially available kits on HITACHI 7020 chemistry analyzer. Serum insulin was measured by commercially available reagent kit. Using fasting glucose and insulin, HOMA-IR was calculated by using the formula: glucose (mmol/L) × insulin (mIU/L)/22.5. Erythrocyte membrane phospholipid fatty acids were measured by gas chromatography, as previously described^35^ and expressed as % of a total of erythrocyte membrane phospholipid fatty acids.

### Statistical analysis

Descriptive statistics included crude comparison in demographic factors and metabolic risk factors between the trial arms and between T2D cases and controls. Also, a treatment effect on each metabolic risk factor was evaluated by repeated-measures ANOVA. Values for each metabolite were log transformed. Metabolites with more than 20% of missing values were excluded from the analysis (111 metabolites). Results from 296 metabolites were available for the analyses. Missing values for metabolites included were assumed to be below the detection limit and imputed with half the observed minimum values.

To investigate changes in metabolomics profiles by interventions, we first calculated the changes of 296 metabolites and took pattern recognition approach of the changes. We conducted PCA for the changes. We examined whether each principal-component scale could separate three intervention arms by running linear regression with adjustment for age, sex and BMI, and then examined which metabolites contributed to the principal component separating the intervention arms. We also conducted PLS-DA, as a supervised method, to determine what metabolites’ changes jointly identified the different intervention arms^36^. In the PLS-DA, we ranked the metabolites based on the VIP scores from the first component generated by PLS-DA, as used previously^37^. Based on PCA and PLS-DA, metabolites responding to intervention were characterized.

A change of each metabolite over time was tested by repeated-measures ANOVA in which treatment effects were tested by time × group interaction term. A significant difference was based on a *P* value (α = 0.05) adjusted for multiple testing for 296 signals by using false-discovery rate correction often referred to as q-values^38^. If a significant difference was indicated, pair-wise comparisons between two groups were similarly performed with repeated-measures ANOVA with α = 0.05/3 = 0.017.

Comparison between T2D patients (trial participants) and healthy controls might have confounding. Thus we used multivariable-adjusted linear regression for each of 296 signals, including a metabolite as an independent variable, and case-control status as a dependent variable, with false-discovery-rate correction for multiple testing. To obtain stable estimates with the limited sample size (53 cases/17 controls), we only modelled age, sex and BMI as covariates.

We additionally examined partial correlation coefficients between changes of CMPF, a known chemical present in fish and potentially related to metabolic risk^22^,^23^,^26^, and changes of the serum n-3 fatty acids and conventional metabolic markers, with adjustment for age, sex, and BMI. Similarly, partial correlation coefficients were also examined cross-sectionally in all participants (healthy controls and baseline measures of the trial participants), with adjustment for age, sex, BMI and disease status.

Statistical analyses for the metabolomics data were conducted by using MetaboAnalyst 3.0, an online comprehensive tool suite for metabolomics data analysis^39^,^40^,^41^ (http://www.metaboanalyst.ca/MetaboAnalyst/faces/home.xhtml). The results of MetaboAnalyst were based on the analysis with R version 3.0.3. Other statistical analyses were based on the Stata (version 13; Stata Corp, College Station, TX, USA) or R (version 3.1.3).

## Additional Information

**How to cite this article**: Zheng, J.-S. *et al*. Serum metabolomics profiles in response to n-3 fatty acids in Chinese patients with type 2 diabetes: a double-blind randomised controlled trial. *Sci. Rep.* **6**, 29522; doi: 10.1038/srep29522 (2016).

## Supplementary Material

Supplementary Information

## Figures and Tables

**Table 1:** Characteristics of the participants included in the metabolomics analyses.

	Trial participants with diabetes			*P*_group_*	Healthy control (n = 17)	*P*_case-controls_*
	Fish oil (n = 18)	Flaxseed oil (n = 18)	Corn oil (n = 17)			
Age, y	63.6 ± 6.13	62.8 ± 12.6	63.4 ± 8.87	0.94	59.8 ± 9.2	0.13
Female, n	9	14	13	0.15	10	0.56
Weight, kg	66.8 ± 11.3	64.5 ± 13.8	65.2 ± 12.8	0.65	58 ± 7.56	0.037
BMI	25.3 ± 3.32	25.3 ± 4.76	26.1 ± 3.9	0.95	21.8 ± 1.84	<0.001
SBP, mmHg	141.7 ± 23.6	140.1 ± 15.6	135.3 ± 22.1	0.19	113.5 ± 12.7	<0.001
DBP, mmHg	79.4 ± 10.5	80.1 ± 10.2	76.4 ± 10.1	0.12	67.9 ± 6.68	<0.001
Heart rate, bpm,	72.0 ± 10.8	72.1 ± 10.2	72.8 ± 14.4	0.84	71.5 ± 7.30	0.93
HDL-C, mmol/L	0.96 ± 0.24	1.11 ± 0.29	1.09 ± 0.18	0.09	1.45 ± 0.32	<0.001
LDL-C, mmol/L	3.02 ± 0.90	2.95 ± 0.72	3.01 ± 1.12	0.72	2.61±0.56	0.038
TC, mmol/L	4.57 ± 0.94	4.53 ± 0.71	4.76 ± 1.05	0.37	4.97 ± 0.80	0.25
TG, mmol/L	1.53 ± 0.82	1.43 ± 0.73	1.90 ± 1.08	0.70	1.16 ± 0.47	0.06
Fasting glucose, mmol/L	8.05 ± 3.48	7.27 ± 2.63	6.92 ± 2.56	0.48	4.64 ± 0.52	<0.001

Abbreviation: SBP, systolic blood pressure; DBP, diastolic blood pressure; HDL-C, high-density lipoprotein cholesterol; LDL-C, low-density lipoprotein cholesterol; TC, total cholesterol; TG, triglycerides. Values are expressed as mean  ±  SD.

^*^P_group_ is for differences between the groups of the trial and *P*_case-controls_ is for differences between trial participants and healthy controls, evaluated by analysis-of-variance for continuous variables or Fisher’s exact test for sex proportion.

**Table 2:** Relative concentrations of selected metabolites among Chinese adults with type 2 diabetes in Wuhan, China: a clinical trial of fish oil and flaxseed oil*.

Metabolites	Time	Fish oil (n = 18)	Flaxseed oil (n = 18)	Corn oil (n = 17)	*P*_time_	*P*_group_	*P*_time×group_
CMPF	Baseline	1.30 ± 0.27	0.79 ± 0.16	0.76 ± 0.16	1.7 × 10^−6^	2.12 × 10^−8^	1.8 × 10^−7 ^†
	Endpoint	5.57 ± 0.60	1.33 ± 0.30	1.11 ± 0.23			
EPA	Baseline	1.07 ± 0.08	0.73 ± 0.10	0.97 ± 0.06	2.0 × 10^−6^	4.5 × 10^−4^	4.6 × 10^−6 ^†,‡
	Endpoint	3.05 ± 0.40	0.87 ± 0.09	0.73 ± 0.07			
PC-EPA	Baseline	0.93 ± 0.10	0.64 ± 0.09	0.83 ± 0.10	1.5 × 10^−4^	1.7 × 10^−4^	3.4 × 10^−4 ^†,‡
	Endpoint	3.44 ± 0.57	1.09 ± 0.14	0.74 ± 0.10			
DHA	Baseline	1.01 ± 0.06	0.88 ± 0.08	0.97 ± 0.08	0.010	0.105	0.001†
	Endpoint	1.55 ± 0.14	0.86 ± 0.08	0.83 ± 0.07			
Linolenate (n-3 or n-6)	Baseline	1.18 ± 0.10	1.00 ± 0.08	1.37 ± 0.11	0.940	0.015	0.005‡
	Endpoint	0.96 ± 0.09	1.16 ± 0.11	0.93 ± 0.79			
DPA (n-3)	Baseline	1.14 ± 0.10	0.91 ± 0.10	1.39 ± 0.13	0.758	0.551	0.021†,‡
	Endpoint	1.38 ± 0.17	0.94 ± 0.10	0.90 ± 0.10			

^*^Data are expressed are mean ± SE. *P* values were based on ANOVA evaluating the three groups and two time points. The metabolites were selected from 296 metabolites. Only these six factors were statistically significant for differences in changes during follow-up, with *P* values < 0.05 after correction for false-discovery rate for multiple comparisons between three groups.

^†^Five metabolites showed significant differences in changes between fish oil group and corn oil group.

^‡^Four metabolites in this table showed significant differences in changes between flaxseed oil group and corn oil group.

Abbreviation: CMPF, 3-carboxy-4-methyl-5-propyl-2-furanpropanoate; EPA, eicosapentaenoate; PC-EPA, 1-eicosapentaenoylglycerophosphocholine; DHA, docosahexaenoate; DPA, docosapentaenoate.
