# Supplementary material for: Serum metabolomics profiles in response to n-3 fatty acids in Chinese patients with type 2 diabetes: a double-blind randomised controlled trial
Source: Sci Rep. 2016 Jul 12;6:29522. doi: 10.1038/srep29522 (PMC4941578; doi:10.1038/srep29522)
Supplement: Supplementary Information [file srep29522-s1.pdf]

Title: Serum metabolomics profiles in response to n-3 fatty acids in Chinese patients with type 2 diabetes: a double-blind randomised controlled trial

Ju-Sheng Zheng, Mei Lin, Fumiaki Imamura, Wenwen Cai, Ling Wang, Jue-Ping Feng, Yue Ruan, Jun Tang, Fenglei Wang, Hong Yang, Duo Li

Supplemental file 1 Supplemental Figures and Tables

Supplemental Figure legends

Supplemental Figure 1 Hierarchical clusters of 30 metabolites selected from contributions to the principal component after principal component analysis. In a linear regression with this component as dependant variable, adjusting for age, sex and BMI, there was a significant difference between the FSO and CO group (*P*=0.006). Based on 30 metabolites with the highest contributions to this principal component (correlation coefficient <-0.083 or >0.092), cluster analysis was conducted for FO vs CO (A) and FSO vs CO (B), respectively.

Supplemental Figure 2 Ranking of serum metabolites in the partial least square discriminant analysis. The ranking was based on the importance in projection (VIP) from the least square discriminant analysis for FO vs CO (A) and FSO vs CO (B), respectively. Red color indicated higher level compared with green color. CMPF, 3-carboxy-4-methyl-5-propyl-2-furanpropanoate; EPA, eicosapentaenoate; DHA, docosahexaenoate; PC-EPA, 1-eicosapentaenoylglycerophosphocholine.

Supplemental Figure 3 Hierarchical clusters of top 30 significant metabolites between healthy controls and type 2 diabetic patients at baseline identified by linear regression. Linear regression was adjusted for age, sex and BMI.

Supplemental Figure 4 Predicted levels of six metabolites in healthy controls and type 2 diabetic patients at baseline. Six metabolites were selected from results from the intervention trial. Levels of the six metabolites: CMPF, PC-EPA, EPA, DHA, linolenate (n-3 or n-6) and DPA (n-3), were projected from a linear regression including the case status, age, sex and BMI. *indicate significant difference (*P*<0.05, with false-discovery-rate correction for multiple testing) for the metabolites between healthy controls and type 2 diabetes cases at baseline in the linear regression model, adjusting for age, sex and BMI. CMPF, 3-carboxy-4-methyl-5-propyl-2-furanpropanoate; EPA, eicosapentaenoate; DHA, docosahexaenoate; PC-EPA, 1-eicosapentaenoylglycerophosphocholine; DPA, docosapentaenoate.

Supplemental Table legends

Supplemental Table 1 Change of blood metabolic markers after the intervention.
*P < 0.05 for the paired t test. †P values were based on two-way ANOVA evaluating the three groups and two time points. HOMA-IR, Homeostasis Model Assessment of Insulin Resistance; HDL-C, high-density lipoprotein cholesterol; LDL-C, low-density lipoprotein cholesterol; TC, total cholesterol; TG, triglycerides.

Supplemental Table 2 Difference between intervention groups for the five components derived from the partial least square-discriminant analysis.
*A linear regression was conducted to identify the difference between groups for the five partial least square-discriminant analysis derived components, adjusting for age, sex and BMI. †The P-values for the first three components were all significant (P<0.05) between fish

oil or flaxseed oil, compared with corn oil. This indicated that all the three components could distinguish fish oil or flaxseed oil from the corn oil group.

Supplemental Table 3 Top 30 significant metabolites between type 2 diabetic cases and healthy controls identified by linear regression.

*Linear regression was adjusted for age, sex and BMI.

A

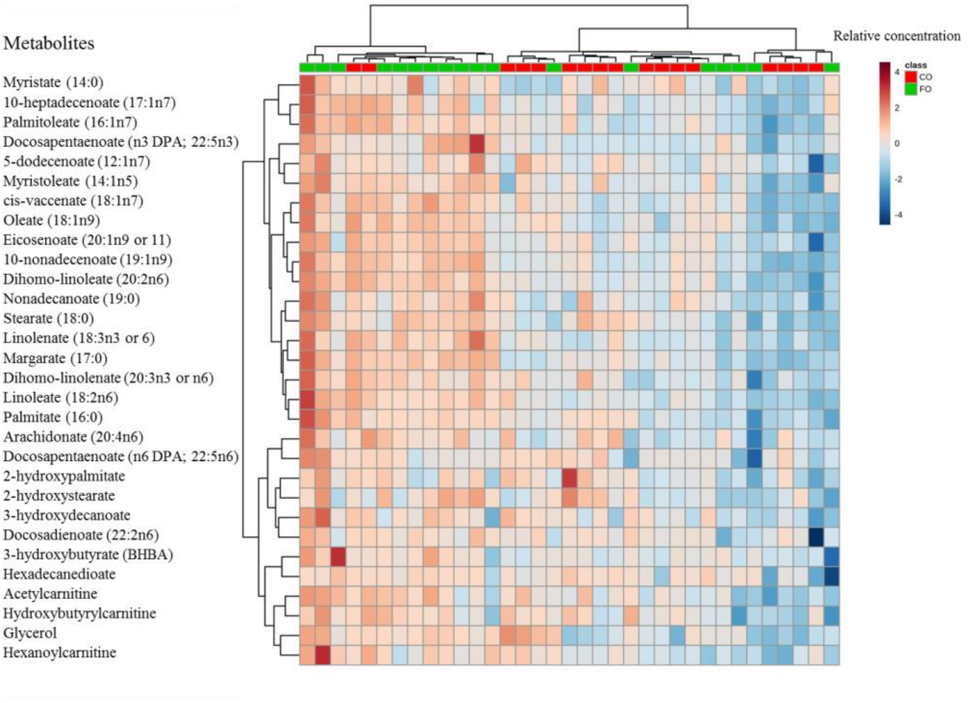

B

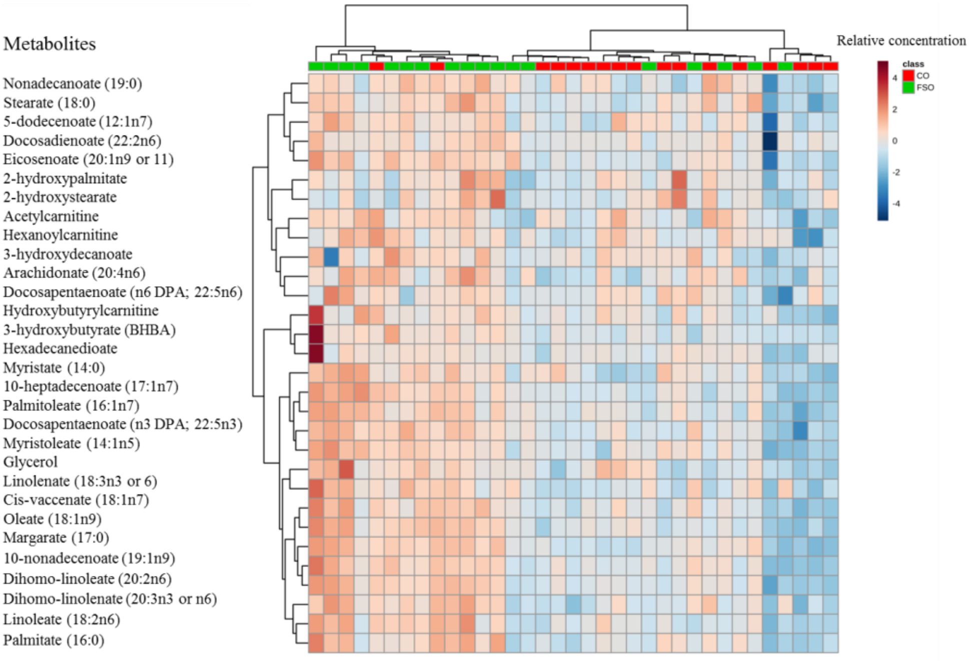

Supplemental Figure 1 Hierarchical clusters of 30 metabolites selected from contributions to the principal component after principal component analysis. In a linear regression with this component as dependant variable, adjusting for age, sex and BMI, there was a significant difference between the FSO and CO group (P=0.006). Based on 30 metabolites with the highest contributions to this principal component (correlation coefficient <-0.083 or >0.092), cluster analysis was conducted for FO vs CO (A) and FSO vs CO (B), respectively.

A

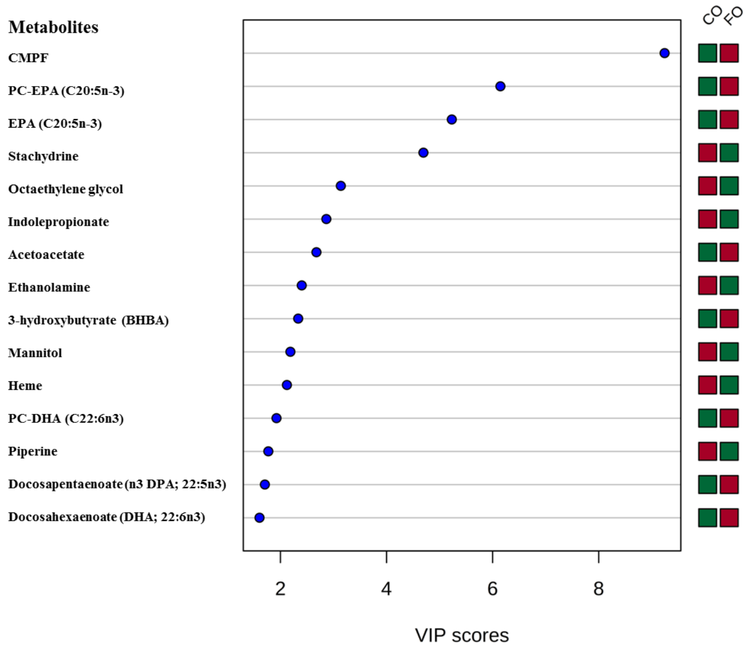

B

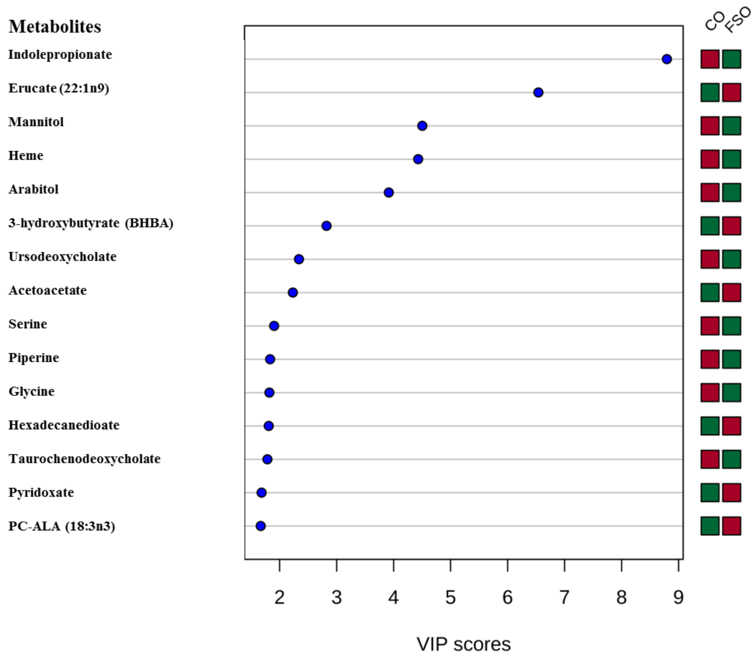

Supplemental Figure 2 Ranking of serum metabolites in the partial least square discriminant analysis. The ranking was based on the importance in projection (VIP) from the least square discriminant analysis for FO vs CO (A) and FSO vs CO (B), respectively. Red color indicated higher level compared with green color. CMPF, 3-carboxy-4-methyl-5-propyl-2-furanpropanoate; EPA, eicosapentaenoate; DHA, docosahexaenoate; PC-EPA, 1-eicosapentaenoylglycerophosphocholine.

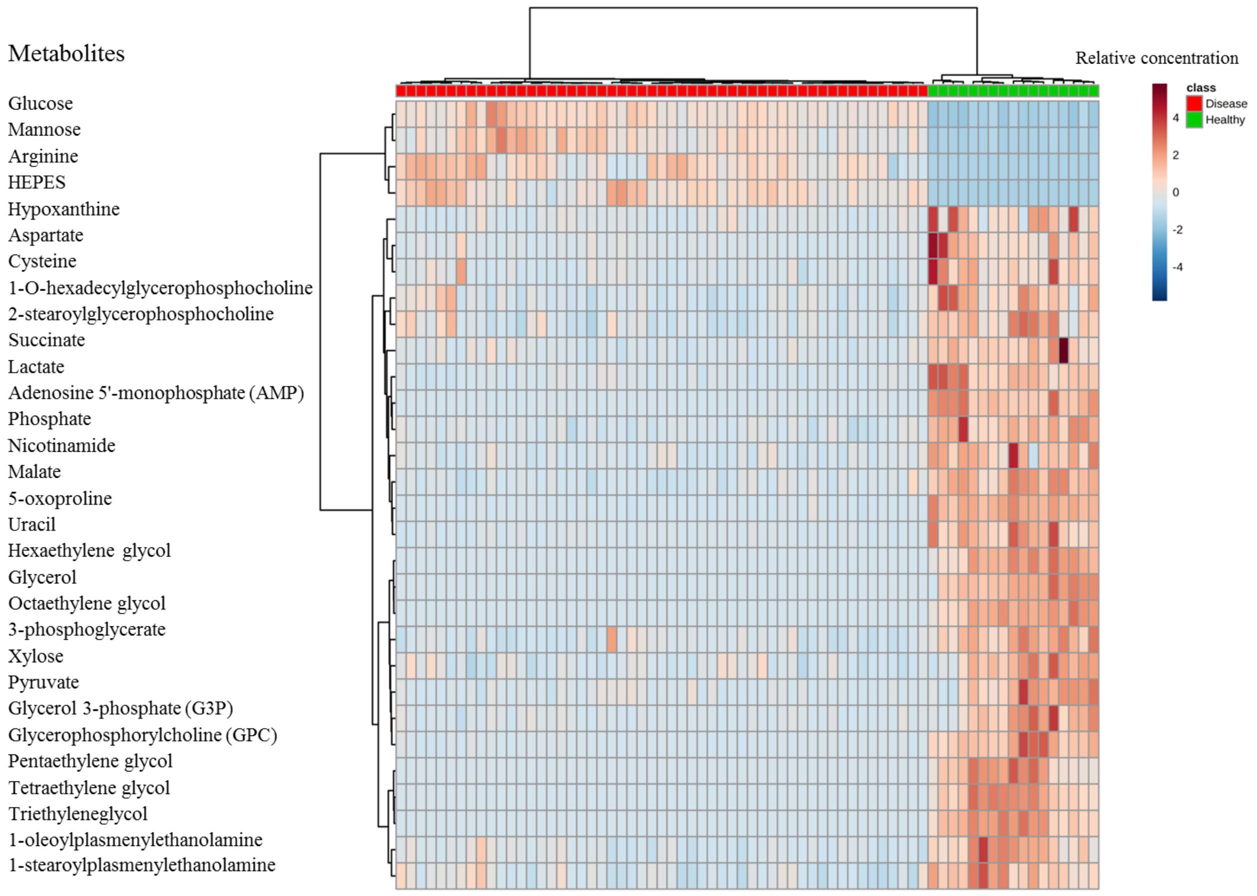

Supplemental Figure 3 Hierarchical clusters of top 30 significant metabolites between healthy controls and type 2 diabetic patients at baseline identified by linear regression. Linear regression was adjusted for age, sex and BMI.

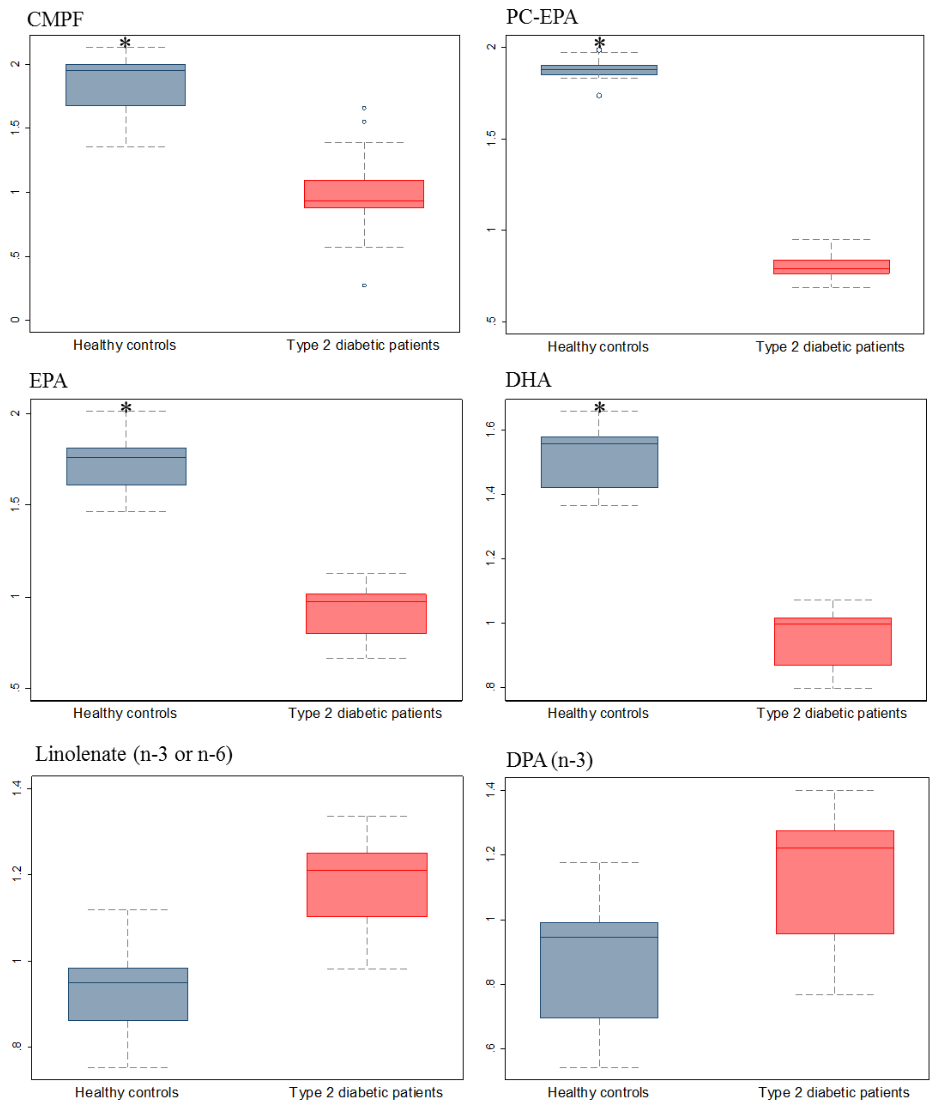

Supplemental Figure 4 Predicted levels of six metabolites in healthy controls and type 2 diabetic patients at baseline. Six metabolites were selected from results from the intervention trial. Levels of the six metabolites: CMPF, PC-EPA, EPA, DHA, linolenate (n-3 or n-6) and DPA (n-3), were projected from a linear regression including the case status, age, sex and BMI. *indicate significant difference (P<0.05, with false-discovery-rate correction for multiple testing) for the metabolites between healthy controls and type 2 diabetes cases at baseline in the linear regression model, adjusting for age, sex and BMI. CMPF, 3-carboxy-4-methyl-5-propyl-2-furanpropanoate; EPA, eicosapentaenoate; DHA, docosahexaenoate; PC-EPA, 1-eicosapentaenoylglycerophosphocholine; DPA, docosapentaenoate.

Supplemental Table 1 Change of blood metabolic markers after the intervention

	Fish oil (FO)			Flaxseed oil (FSO)			Corn oil (CO)			P_{time}†	P_{group}†	$P_{time×group}$†
	Day 0	Day 180	Difference	Day 0	Day 180	Difference	Day 0	Day 180	Difference			
Glucose, mmol/L	8.05±3.48	8.52±2.80	0.47(-1.83,2.78)	7.26±2.53	7.50±1.98	0.24(-0.79,1.27)	7.01±2.44	7.86±2.34	0.85(0.18,1.52)*	0.82	0.11	0.92
Insulin, mU/L	15.0±9.09	14.3±7.3	-0.70(-3.51,2.11)	19.3±23.0	16.6±12.2	-2.74(-9.01,3.54)	17.2±23.5	16.9±15.1	-0.29(-6.18,5.61)	0.88	0.30	0.71
HOMA-IR	5.55±4.15	5.70±4.12	0.15(-1.63,1.93)	6.62±7.50	5.94±5.37	-0.68(-2.97,1.61)	5.32±7.23	6.06±5.68	0.74(-1.26,2.74)	0.88	0.09	0.71
HDL-C, mmol/L	0.96±0.24	1.12±0.35	0.16(0.08,0.24)*	1.10±0.28	1.17±0.24	0.07(-0.02,0.16)	1.13±0.20	1.26±0.24	0.13(0.06,0.19)*	0.52	$7.52×10^{-5}$	0.71
LDL-C, mmol/L	3.02±0.90	2.88±0.90	-0.14(-0.58,0.30)	3.03±0.75	2.94±0.76	-0.09(-0.31,0.14)	3.19±1.02	3.09±0.98	-0.10(-0.44,0.24)	0.88	0.36	0.92
TC, mmol/L	4.57±0.94	4.50±1.07	-0.07(-0.48,0.34)	4.59±0.76	4.81±0.93	0.22(-0.02,0.47)	4.91±0.99	4.97±1.14	-0.06(-0.37,0.50)	0.83	0.64	0.71
TG, mmol/L	1.53±0.82	1.01±0.44	-0.52(-0.77,-0.28)*	1.44±0.71	1.67±0.90	0.23(-0.05,0.50)	1.67±0.73	1.30±0.77	-0.36(-0.62,-0.11)*	0.83	$7.52×10^{-5}$	$7.27×10^{-5}$

*P < 0.05 for the paired t test.

†P values were based on two-way ANOVA evaluating the three groups and two time points. HOMA-IR, Homeostasis Model Assessment of Insulin Resistance; HDL-C, high-density lipoprotein cholesterol; LDL-C, low-density lipoprotein cholesterol; TC, total cholesterol; TG, triglycerides.

Supplemental Table 2 Difference between intervention groups for the five components derived from the partial least square-discriminant analysis

		**Fish oil vs Corn oil**			**Flaxseed oil vs Corn oil**		
PLS components	Variance explained	Beta coefficient*	SE	*P*†	Beta coefficient*	SE	*P*†
Components 1	12.5%	1.06 (0.50, 1.62)	0.28	<0.001	1.64 (1.10, 2.17)	0.27	<0.001
Components 2	10.5%	1.11 (0.56, 1.66)	0.27	<0.001	1.68 (1.15, 2.21)	0.26	<0.001
Components 3	6.6%	0.82 (0.20, 1.43)	0.31	0.01	1.32 (0.73, 1.91)	0.29	<0.001
Components 4	10.7%	0.42 (-0.20, 1.05)	0.31	0.181	1.10 (0.50, 1.70)	0.3	0.001
Components 5	4.7%	0.48 (-0.18, 1.15)	0.33	0.149	1.03 (0.39, 1.66)	0.32	0.002

*A linear regression was conducted to identify the difference between groups for the five partial least square-discriminant analysis derived components, adjusting for age, sex and BMI.

†The *P*-values for the first three components were all significant ($P<0.05$) between fish oil or flaxseed oil, compared with corn oil. This indicated that all the three components could distinguish fish oil or flaxseed oil from the corn oil group.

Supplemental Table 3 Top 30 significant metabolites between type 2 diabetic cases and healthy controls identified by linear regression

Name	Super pathway	Sub pathway	FDR corrected *P*-value*	Means in Cases	SD	Means in controls	SD
5-oxoproline	Amino Acid	Glutathione Metabolism	1.15E-40	1.014	0.165	4.471	0.617
Octaethylene glycol	Xenobiotics	Chemical	2.78E-27	1.129	0.776	392.294	131.347
Adenosine 5'-monophosphate (AMP)	Nucleotide	Purine Metabolism, Adenine containing	4.30E-26	0.817	0.589	22.450	7.853
Triethyleneglycol	Xenobiotics	Chemical	1.96E-24	1.063	0.599	172.614	64.411
Glycerol	Lipid	Glycerolipid Metabolism	2.32E-24	1.019	0.296	20.848	7.760
Hexaethylene glycol	Xenobiotics	Chemical	8.71E-24	0.982	0.362	65.041	25.608
Malate	Energy	TCA Cycle	7.44E-23	1.026	0.288	3.255	0.751
Tetraethylene glycol	Xenobiotics	Chemical	5.23E-21	0.916	0.459	295.795	132.464
Uracil	Nucleotide	Pyrimidine Metabolism, Uracil containing	1.19E-19	0.872	0.307	6.422	2.628
Glycerophosphorylcholine (GPC)	Lipid	Phospholipid Metabolism	1.26E-19	0.833	0.289	7.250	2.938
Lactate	Carbohydrate	Glycolysis, Gluconeogenesis, and Pyruvate Metabolism	2.02E-19	0.902	0.227	3.791	1.395
Phosphate	Energy	Oxidative Phosphorylation	2.02E-19	0.956	0.126	2.069	0.478
Glucose	Carbohydrate	Glycolysis, Gluconeogenesis, and Pyruvate Metabolism	5.19E-19	1.097	0.248	0.160	0.096
HEPES	Xenobiotics	Chemical	1.11E-16	1.113	0.153	0.670	0.000
Pentaethylene glycol	Xenobiotics	Chemical	4.17E-16	0.974	0.204	80.175	45.116
3-phosphoglycerate	Carbohydrate	Glycolysis, Gluconeogenesis, and Pyruvate Metabolism	5.33E-15	0.791	0.671	3.583	1.258
Arginine	Amino Acid	Urea cycle; Arginine and Proline Metabolism	8.25E-15	0.944	0.283	0.284	0.200
Nicotinamide	Cofactors and Vitamins	Nicotinate and Nicotinamide Metabolism	2.00E-13	0.999	0.530	4.605	2.155
1-oleoylplasmenylethanolamine	Lipid	Lysolipid	2.30E-13	0.907	0.408	3.302	1.386
1-stearoylplasmenylethanolamine	Lipid	Lysolipid	2.03E-12	0.940	0.401	2.470	0.757
Glycerol 3-phosphate (G3P)	Lipid	Glycerolipid Metabolism	2.23E-12	0.924	0.224	3.340	1.695
Xylose	Carbohydrate	Pentose Metabolism	3.32E-12	0.986	0.262	2.106	0.691

Mannose	Carbohydrate	Fructose, Mannose and Galactose Metabolism	4.33E-12	1.093	0.381	0.109	0.060
Succinate	Energy	TCA Cycle	1.84E-11	0.934	0.252	2.565	1.194
Pyruvate	Carbohydrate	Glycolysis, Gluconeogenesis, and Pyruvate Metabolism	3.29E-11	1.028	0.325	3.596	1.888
Aspartate	Amino Acid	Alanine and Aspartate Metabolism	1.51E-10	1.225	0.657	6.562	4.464
Cysteine	Amino Acid	Methionine, Cysteine, SAM and Taurine Metabolism	3.59E-10	0.833	0.417	2.526	1.310
1-O-hexadecylglycerophosphocholine	Lipid	Lysolipid	1.03E-09	1.051	0.466	2.727	1.091
2-stearoylglycerophosphocholine	Lipid	Lysolipid	3.69E-09	1.044	0.418	2.360	0.811
Hypoxanthine	Nucleotide	Purine Metabolism, (Hypo)Xanthine/Inosine containing	6.95E-09	1.076	0.528	4.459	3.123

*Linear regression was adjusted for age, sex and BMI.

Supplemental file 2 Data quality and introduction of Metabolon platform

Data Quality: Instrument and Process Variability

Supplemental Table 1 Instrument and process variability

QC Sample	***Measurement***	***Median RSD***
**Internal Standards**	Instrument Variability	5 %
**Endogenous Biochemicals**	Total Process Variability	11 %

Instrument variability was determined by calculating the median relative standard deviation (RSD) for the internal standards that were added to each sample prior to injection into the mass spectrometers. Overall process variability was determined by calculating the median RSD for all endogenous metabolites (i.e., non-instrument standards) present in 100% of the Client Matrix samples, which are technical replicates of pooled client samples. Values for instrument and process variability as shown in the table above meet Metabolon's acceptance criteria (**Supplemental Table 1**).

Metabolon Platform

Sample Accessioning: Following receipt, samples were inventoried and immediately stored at -80°C. Each sample received was accessioned into the Metabolon LIMS system and was assigned by the LIMS a unique identifier that was associated with the original source identifier only. This identifier was used to track all sample handling, tasks, results, etc. The samples (and all derived aliquots) were tracked by the LIMS system. All portions of any sample were automatically assigned their own unique identifiers by the LIMS when a new task was created; the relationship of these samples was also tracked. All samples were maintained at -80°C until processed.

Sample Preparation: Samples were prepared using the automated MicroLab STAR® system from Hamilton Company. A recovery standard was added prior to the first step in the extraction process for QC purposes. To remove protein, dissociate small molecules bound to protein or trapped in the precipitated protein matrix, and to recover chemically diverse metabolites, proteins were precipitated with methanol under vigorous shaking for 2 min (Glen Mills GenoGrinder 2000) followed by centrifugation. The resulting extract was divided into five fractions: one for analysis by UPLC-MS/MS with positive ion mode electrospray ionization, one for analysis by UPLC-MS/MS with negative ion mode electrospray ionization, one for LC polar platform, one for analysis by GC-MS, and one sample was reserved for backup. Samples were placed briefly on a TurboVap® (Zymark) to remove the organic solvent. For LC, the samples were stored overnight under nitrogen before preparation for analysis. For GC, each sample was dried under vacuum overnight before preparation for analysis.

QA/QC: Several types of controls were analyzed in concert with the experimental samples: a pooled matrix sample generated by taking a small volume of each experimental sample (or alternatively, use of a pool of well-characterized human plasma) served as a technical replicate throughout the data set; extracted water samples served as process blanks; and a cocktail of QC standards that were carefully chosen not to interfere with the measurement of endogenous compounds were spiked into every analyzed sample, allowed instrument performance monitoring and aided chromatographic alignment. Supplemental Tables 2 and 3 describe these QC samples and standards. Instrument variability was determined by calculating the median relative standard deviation (RSD) for the standards that were added to each sample prior to injection into the mass spectrometers. Overall process variability was determined by calculating the median RSD for all endogenous metabolites (i.e., non-instrument standards) present in 100% of the pooled matrix samples. Experimental samples were randomized across the platform run with QC samples spaced evenly among the injections, as outlined in Supplemental Figure 1.

Supplemental Table 2 Description of Metabolon QC Samples

Type	Description	Purpose
**MTRX**	Large pool of human plasma maintained by Metabolon that has been characterized extensively.	**Assure that all aspects of the Metabolon process are operating within specifications.**
**CMTRX**	Pool created by taking a small aliquot from every customer sample.	**Assess the effect of a non-plasma matrix on the Metabolon process and distinguish biological variability from process variability.**
**PRCS**	Aliquot of ultra-pure water	**Process Blank used to assess the contribution to compound signals from the process.**
**SOLV**	**Aliquot of solvents used in extraction.**	**Solvent Blank used to segregate contamination sources in the extraction.**

Supplemental Table 3 Metabolon QC Standards

Type	Description	Purpose
**RS**	Recovery Standard	**Assess variability and verify performance of extraction and instrumentation.**
**DS**	Derivatization Standard	**Assess variability of derivatization for GC-MS samples.**
**IS**	**Internal Standard**	**Assess variability and performance of instrument.**

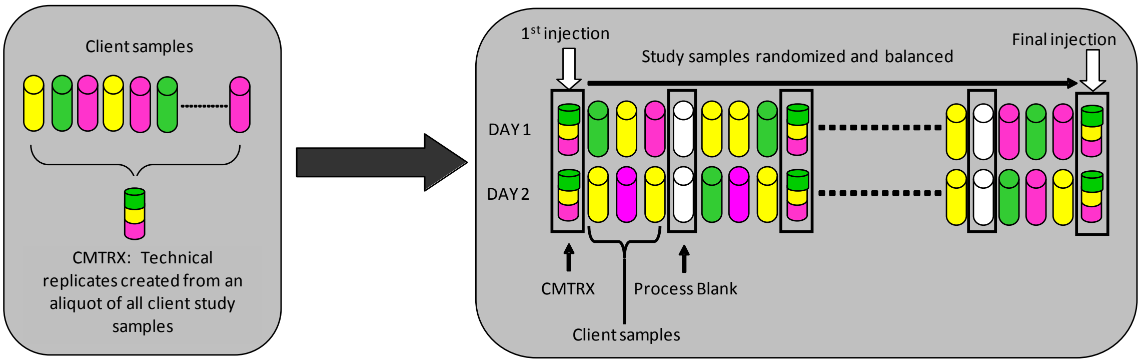

Supplemental Figure 1 Preparation of client-specific technical replicates. A small aliquot of each client sample (colored cylinders) is pooled to create a CMTRX technical replicate sample (multi-colored cylinder), which is then injected periodically throughout the platform run. Variability among consistently detected biochemicals can be used to calculate an estimate of overall process and platform variability.

Ultrahigh Performance Liquid Chromatography-Tandem Mass Spectroscopy (UPLC-MS/MS): The LC/MS portion of the platform was based on a Waters ACQUITY ultra-performance liquid chromatography (UPLC) and a Thermo Scientific Q-Exactive high resolution/accurate mass spectrometer interfaced with a heated electrospray ionization (HESI-II) source and Orbitrap mass analyzer operated at 35,000 mass resolution. The sample extract was dried then reconstituted in acidic or basic LC-compatible solvents, each of which contained 8 or more injection standards at fixed concentrations to ensure injection and chromatographic consistency. One aliquot was analyzed using acidic positive ion optimized conditions and the other using basic negative ion optimized conditions in two independent injections using separate dedicated columns (Waters UPLC BEH C18-2.1x100 mm, 1.7 µm). Extracts reconstituted in acidic conditions were gradient eluted from a C18 column using water and methanol containing 0.1% formic acid. The basic extracts were similarly eluted from C18 using methanol and water, however with 6.5mM Ammonium Bicarbonate. The third aliquot was analyzed via negative ionization following elution from a HILIC column (Waters UPLC BEH Amide 2.1x150 mm, 1.7 µm) using a gradient consisting of water and acetonitrile with 10mM Ammonium Formate. The MS analysis alternated between MS and data-dependent MS2 scans using dynamic exclusion, and the scan range was from 80-1000 m/z. Raw data files are archived and extracted as described below.

Gas Chromatography-Mass Spectroscopy (GC-MS): The samples destined for analysis by GC-MS were dried under vacuum for a minimum of 18 h prior to being derivatized under dried nitrogen using bistrimethyl-silyltrifluoroacetamide. Derivatized samples were separated on a 5% diphenyl / 95% dimethyl polysiloxane fused silica column (20 m x 0.18 mm ID; 0.18 um film thickness) with helium as carrier gas and a temperature ramp from 60° to 340°C in a 17.5 min period. Samples were analyzed on a Thermo-Finnigan Trace DSQ fast-scanning single-quadrupole mass spectrometer using electron impact ionization (EI) and operated at unit mass resolving power. The scan range was from 50–750 m/z. Raw data files are archived and extracted as described below.

Bioinformatics: The informatics system consisted of four major components, the Laboratory Information Management System (LIMS), the data extraction and peak-identification software, data processing tools for QC and compound identification, and a collection of information interpretation and visualization tools for use by data analysts. The hardware and software foundations for these informatics components were the LAN backbone, and a database server running Oracle 10.2.0.1 Enterprise Edition.

LIMS: The purpose of the Metabolon LIMS system was to enable fully auditable laboratory automation through a secure, easy to use, and highly specialized system. The scope of the Metabolon LIMS system encompasses sample accessioning, sample preparation and instrumental analysis and reporting and advanced data analysis. All of the subsequent software systems are grounded in the LIMS data structures. It has been modified to leverage and interface with the in-house information extraction and data visualization systems, as well as third party instrumentation and data analysis software.

Data Extraction and Compound Identification: Raw data was extracted, peak-identified and QC processed using Metabolon’s hardware and software. These systems are built on a web-service platform utilizing Microsoft’s. NET technologies, which run on high-performance application servers and fiber-channel storage arrays in clusters to provide active failover and load-balancing. Compounds were identified by comparison to library entries of purified standards or recurrent unknown entities. Metabolon maintains a library based on authenticated standards that contains the retention time/index (RI), mass to charge ratio (*m/z)*,

and chromatographic data (including MS/MS spectral data) on all molecules present in the library. Furthermore, biochemical identifications are based on three criteria: retention index within a narrow RI window of the proposed identification, accurate mass match to the library +/- 0.005 amu, and the MS/MS forward and reverse scores between the experimental data and authentic standards. The MS/MS scores are based on a comparison of the ions present in the experimental spectrum to the ions present in the library spectrum. While there may be similarities between these molecules based on one of these factors, the use of all three data points can be utilized to distinguish and differentiate biochemicals. More than 3300 commercially available purified standard compounds have been acquired and registered into LIMS for distribution to both the LC-MS and GC-MS platforms for determination of their analytical characteristics. Additional mass spectral entries have been created for structurally unnamed biochemicals, which have been identified by virtue of their recurrent nature (both chromatographic and mass spectral). These compounds have the potential to be identified by future acquisition of a matching purified standard or by classical structural analysis.

Curation: A variety of curation procedures were carried out to ensure that a high quality data set was made available for statistical analysis and data interpretation. The QC and curation processes were designed to ensure accurate and consistent identification of true chemical entities, and to remove those representing system artifacts, mis-assignments, and background noise. Metabolon data analysts use proprietary visualization and interpretation software to confirm the consistency of peak identification among the various samples. Library matches for each compound were checked for each sample and corrected if necessary.

Metabolite Quantification and Data Normalization: Peaks were quantified using area-under-the-curve. For studies spanning multiple days, a data normalization step was performed to correct variation resulting from instrument inter-day tuning differences. Essentially, each compound was corrected in run-day blocks by registering the medians to equal one (1.00) and normalizing each data point proportionately (termed the "block correction"; Supplemental Figure 2). For studies that did not require more than one day of analysis, no normalization is necessary, other than for purposes of data visualization. In certain instances, biochemical data may have been normalized to an additional factor (e.g., cell counts, total protein as determined by Bradford assay, osmolality, etc.) to account for differences in metabolite levels due to differences in the amount of material present in each sample.

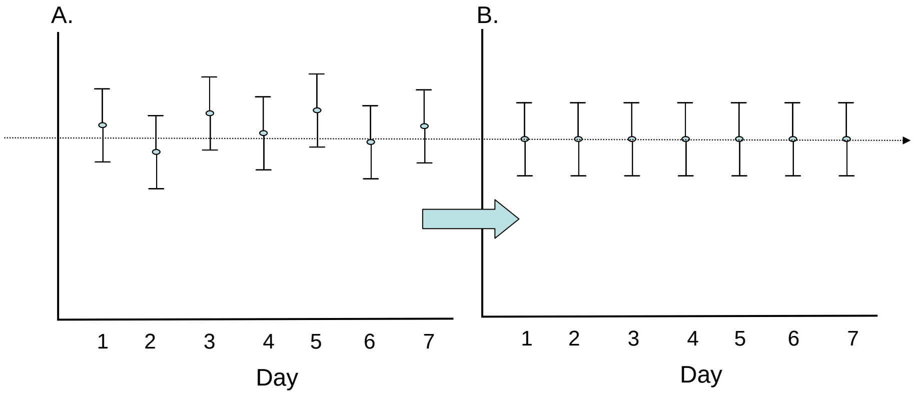

Supplemental Figure 2 Visualization of data normalization steps for a multiday platform run.
